# Supplementary material for: A whole-animal phenotypic drug screen identifies suppressors of atherogenic lipoproteins
Source: eLife. 2026 Aug 27;14:RP105314. doi: 10.7554/eLife.105314 (PMC13521566; doi:10.7554/eLife.105314)
Supplement: Supplementary file 2 [file elife-105314-code1.zip › JHDL_primary_result/MEDROXYPROGESTERONE ACETATE/MEDROXYPROGESTERONE ACETATE_JHDL_4_008_008_2019_05_10_11_14.pdf]

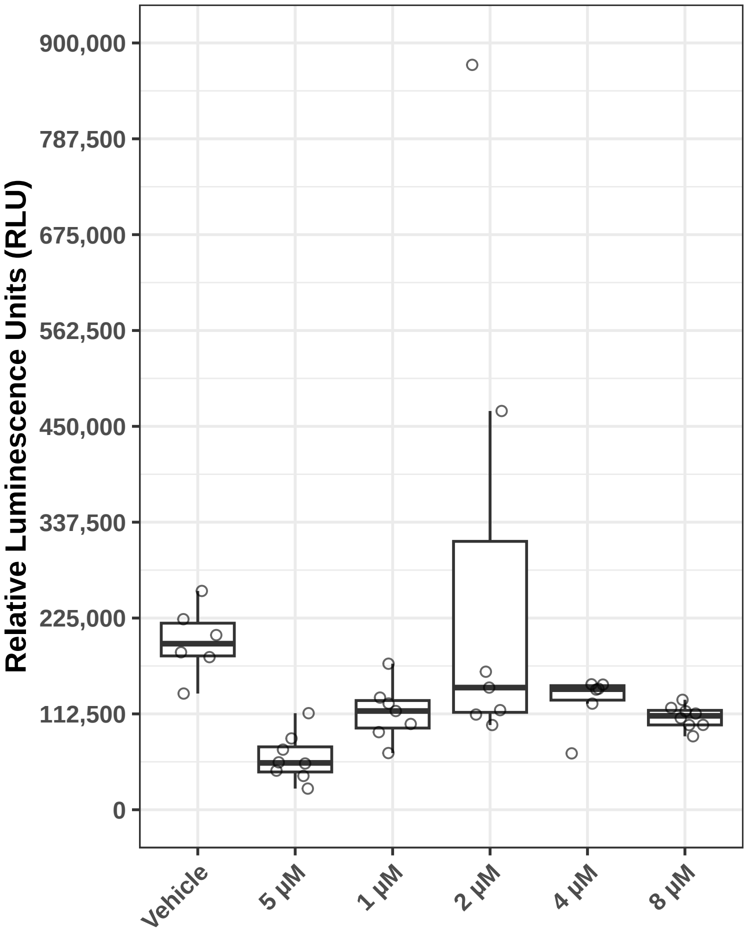

Relative Luminescence Units (RLU)
900,000
787,500
675,000
562,500
450,000
337,500
225,000
112,500
0
Vehicle
5 μM
1 μM
2 μM
4 μM
8 μM
